# Mechanistic study of the differences in lactic acid bacteria resistance to freeze- or spray-drying and storage

**DOI:** 10.1007/s00253-024-13186-3

**Published:** 2024-06-05

**Authors:** Maite Gagneten, Stéphanie Passot, Stéphanie Cenard, Sarrah Ghorbal, Carolina Schebor, Fernanda Fonseca

**Affiliations:** 1 Departamento de Industrias, Facultad de Ciencias Exactas y Naturales, ITAPROQ (UBA- CONICET), Universidad de Buenos Aires, Ciudad Autónoma de Buenos Aires, Argentina; 2 Université Paris-Saclay, INRAE, AgroParisTech, UMR SayFood, Palaiseau, F-91120 France

**Keywords:** *Lb. bulgaricus*, *Lpb. plantarum*, Drying, FTIR spectroscopy, Glass transition temperature, Activation energy

## Abstract

**Abstract:**

*Lactobacillus delbrueckii* subsp. *bulgaricus* and *Lactiplantibacillus plantarum* are two lactic acid bacteria (LAB) widely used in the food industry. The objective of this work was to assess the resistance of these bacteria to freeze- and spray-drying and study the mechanisms involved in their loss of activity. The culturability and acidifying activity were measured to determine the specific acidifying activity, while membrane integrity was studied by flow cytometry. The glass transitions temperature and the water activity of the dried bacterial suspensions were also determined. Fourier transform infrared (FTIR) micro-spectroscopy was used to study the biochemical composition of cells in an aqueous environment. All experiments were performed after freezing, drying and storage at 4, 23 and 37 °C. The results showed that *Lb. bulgaricus* CFL1 was sensitive to osmotic, mechanical, and thermal stresses, while *Lpb. plantarum* WCFS1 tolerated better the first two types of stress but was more sensitive to thermal stress. Moreover, FTIR results suggested that the sensitivity of *Lb. bulgaricus* CFL1 to freeze-drying could be attributed to membrane and cell wall degradation, whereas changes in nucleic acids and proteins would be responsible of heat inactivation of both strains associated with spray-drying. According to the activation energy values (47–85 kJ/mol), the functionality loss during storage is a chemically limited reaction. Still, the physical properties of the glassy matrix played a fundamental role in the rates of loss of activity and showed that a glass transition temperature 40 °C above the storage temperature is needed to reach good preservation during storage.

**Key points:**

• *Specific FTIR bands are proposed as markers of osmotic, mechanic and thermal stress*

• *Lb. bulgaricus CFL1 was sensitive to all three stresses, Lpb. plantarum WCFS1 to thermal stress only*

• *Activation energy revealed chemically limited reactions ruled the activity loss in storage*

**Graphical abstract:**

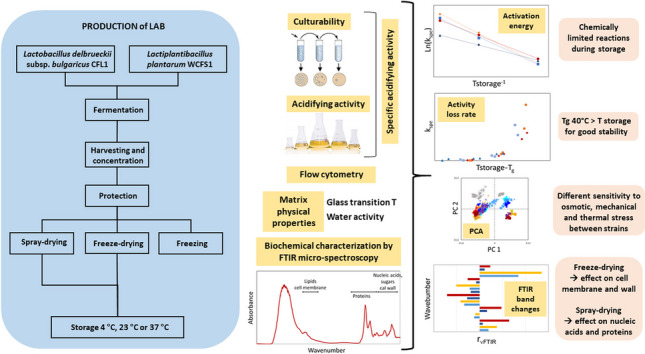

**Supplementary Information:**

The online version contains supplementary material available at 10.1007/s00253-024-13186-3.

## Introduction

Lactic acid bacteria (LAB) concentrates are widely used in the food and health industries to produce a variety of fermented products, and probiotics. Freezing is one of the most employed LAB preservation techniques. However, it entails very low storage temperatures (commonly − 80 °C), which require the right infrastructure and comprises high energy consumption (Broeckx et al. 2016; Pénicaud et al. 2018). Drying the LAB starter cultures is an interesting stabilization strategy to obtain products with longer shelf-life at higher, even non-sub-zero temperatures. Yet, the drying operation itself might cause damage and loss of viability in bacterial populations (Ermis 2021; Santivarangkna et al. 2008; Sehrawat et al. 2022; Yoha et al. 2020a). Freeze-drying is the most commonly used drying method for LAB preservation since it has been shown to lead to higher survival rates than most other drying techniques (Ermis 2021; Kiepś and Dembczyński 2022; Montel Mendoza et al. 2014; Sehrawat et al. 2022; Yoha et al. 2020b). However, it is an energy-intensive, time-consuming, and expensive process (Champagne et al. 2012). Spray-drying is preferred in the food industry for its lower costs and higher productivity when compared to freeze-drying. However, the application of spray-drying to preserve LAB is still limited since some challenges to maintaining cell viability remain unsolved (Huang et al. 2017; Sharma et al. 2022).

During freeze-drying, the temperature decrease exposes cells to cold stress, while ice formation induces osmotic stress because of solute concentration. Mechanical stress is also present in the desorption stage when water is removed by breaking hydrogen bonds. On the other hand, during spray-drying, cells are also exposed to the osmotic and mechanical stresses related to water removal, and additional thermal stress as a consequence of the high operating temperatures employed (Liu et al. 2019; Santivarangkna et al. 2008). The osmotic stress, due to high solute concentration and loss of cell turgor, is known as a major source of cellular damage (Broeckx et al. 2016; Sehrawat et al. 2022). The mechanical stress, associated with water removal, can cause loss of the lipid bilayer integrity and alteration of cell membrane functions (Perdana et al. 2013). The thermal stress due to exposure to high temperatures may cause protein denaturation and aggregation, and damage to essential structures such as ribosomes, nucleic acids, enzymes, and cell membranes (Arena et al. 2019; Capozzi et al. 2012; Perdana et al. 2013; Sehrawat et al. 2022). Also, lipidic components of the cell’s membrane are susceptible to oxidation, which can increase during spray-drying as a consequence of the large surface in contact with air at high temperatures (Broeckx et al. 2016). Therefore, since cell damage caused by stabilization processes can be considered a combination of these different stresses depending on the drying method (Kiepś and Dembczyński 2022; Sehrawat et al. 2022), identifying which kind of stress the different LAB are more sensitive to is essential and could contribute to design the most suitable dehydrating process accordingly.

To improve the cell’s preservation during stabilization and storage, the addition of protective molecules to cell concentrates is applied. These molecules often include mixtures of sugars and polymers that contribute to maintaining cell integrity during freezing, drying and subsequent storage by two proposed mechanisms (Boafo et al. 2022; Crowe et al. 1988; Grasmeijer et al. 2013): (i) the water replacement theory and ii) the vitrification theory. The first one proposes that disaccharides (such as sucrose and trehalose) protect cells during drying by forming hydrogen bonds with membrane polar groups as water is removed, allowing the maintenance of a pseudo-hydrate structure. According to the second theory, the immobilization of the bacteria in a glassy matrix reduces molecular mobility and, consequently, slows down degradation reactions during storage (Liu et al., 2019).

This work aimed to evaluate and compare different dehydration processes and storage conditions to preserve LAB and understand the mechanisms involved in their loss of activity. Using complementary analytical methods and, in particular, an original FTIR micro-spectroscopy method which enables the study of bacterial cells in aqueous conditions while avoiding dehydration that may affect cellular structures, this work seeks to contribute to identify the effect of freeze-drying and spray-drying over the specific cellular components. This FTIR method, previously used to investigate the impact of freezing and freeze-drying processes on LAB (Girardeau et al. 2022; Guerrero et al. 2022, 2023), was here applied for the first time to study the spray-drying process and compare it to freeze-drying. For this purpose, two LAB strains exhibiting different sensitivity to environmental conditions were stabilized with a protective solution containing maltodextrin, fructo-oligosaccharides (FOS) and an antioxidant, and dehydrated by freeze- or spray-drying. *Lactobacillus delbrueckii* subsp. *bulgaricus* CFL1 is known to be very sensitive to drying (Passot et al. 2012), while *Lactiplantibacillus plantarum* WCFS1 has shown more resistance to dehydration (Bensch et al. 2014; G-Alegría et al. 2004; Golowczyc et al. 2010). The FOS present in the protective mixture, not only provides prebiotic functions (Gibson et al. 2004; Rajam and Anandharamakrishnan 2015) but also contains small sugars that can replace water during drying (Romano et al. 2016), while maltodextrin contributes to increase the glass transition temperature of the matrix. To study the LAB biological activity, biochemical and physical characteristics a variety of techniques were applied. Their functional quality was assessed by culturability and acidifying activity measurements, and their membrane integrity by flow cytometry. FTIR micro-spectroscopy was applied to observe differences in the biochemical composition between both LAB and its changes during freeze- and spray-drying and storage. Differential scanning calorimetry and water activity measurements were used to determine the glass transition temperature and the thermodynamic activity of water, crucial parameters affecting the stability of dried products (Guerrero Sanchez et al. 2022, 2023; Roos 2010).

## Materials and methods

**Fig. 1:**
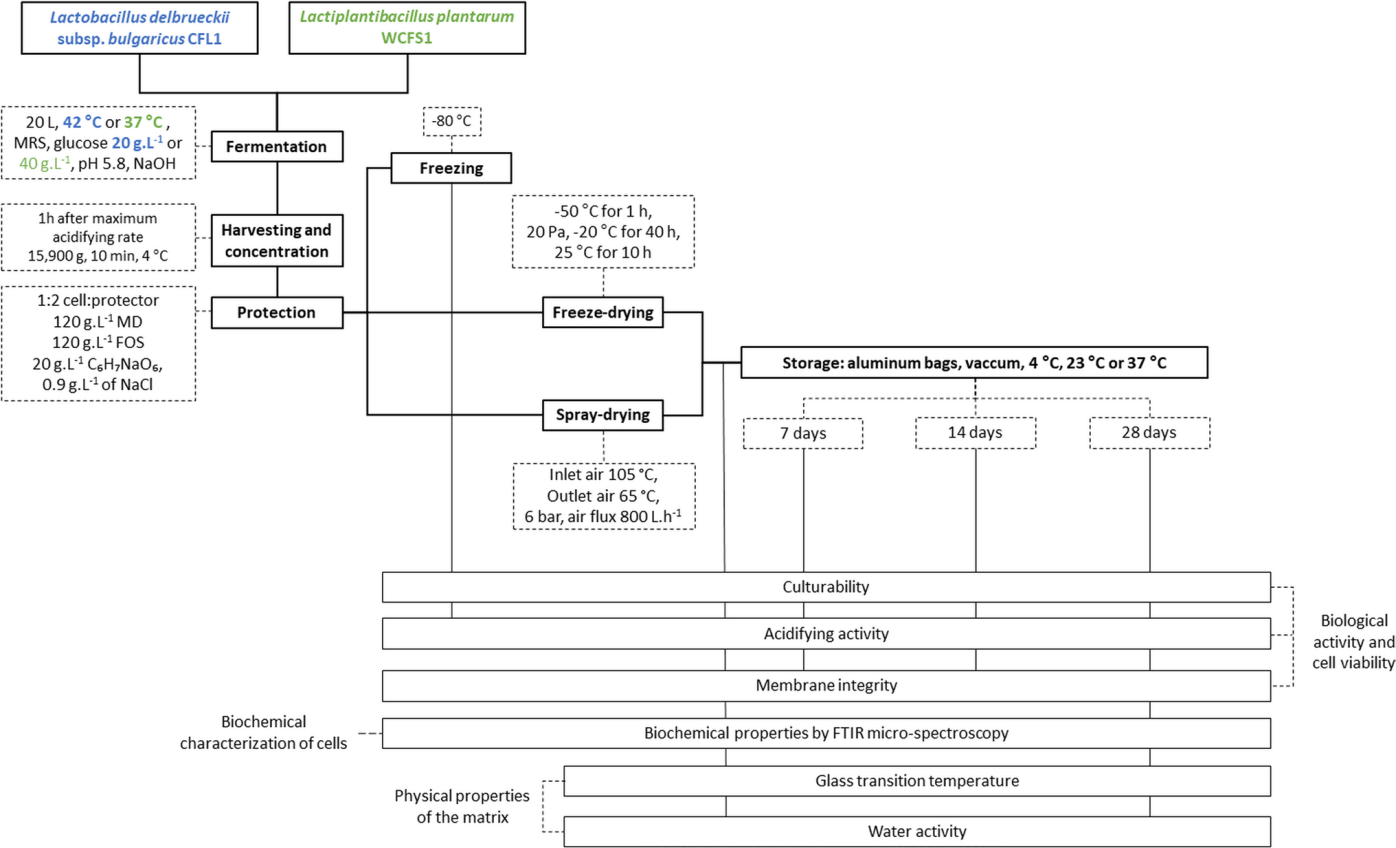
Diagram of the experimental approach and the main parameters investigated in this study. MD: maltodextrin, FOS: fructo-oligosaccharides

The experimental approach followed in this study and the main parameters analyzed are summarized in Fig. 1.

### Production of bacteria

#### Bacterial strains and culture conditions

*Lactobacillus delbrueckii* subsp. *bulgaricus* CFL1 (CIRM-BIA; Rennes, France) and *Lactiplantibacillus plantarum* WCFS1 (NIZO Food Research B.V., The Netherlands) were chosen for this work due to their industrial relevance in the food industry.

Inocula were precultured twice before inoculating the bioreactor. The detailed information of preculture and fermentation conditions for each microorganism are provided elsewhere (10.57745/AAARUZ).

The second preculture of *Lb. bulgaricus* CFL1 and *Lpb. plantarum* WCFS1was used to inoculate a 20 L bioreactor containing 15 L of sterilized (121 °C for 20 min) MRS broth complemented with additional 20–40 g.L^−1^ of glucose, respectively. The temperature was maintained at 42 °C for *Lb*. *bulgaricus* CFL1 and 37 °C *Lpb. plantarum* WCFS1. The pH was maintained at 5.8 with a NaOH solution (17 and 20% v/v, respectively). The consumption of NaOH was monitored and used to calculate the acidifying rate. Cells were harvested by centrifugation (15,900 g, 10 min, 4 °C) one hour after the maximum acidifying rate was reached. Concentrated cells were re-suspended at 4 °C in a 1:2 cells: protective medium ratio (g/g). The protective medium consisted of 120 g.L^−1^ of a commercial maltodextrin (Glucidex 6®, dextrose equivalent of 6, Roquette; Lestrem, France), 120 g.L^−1^ of a commercial mixture of fructo-oligosaccharides (Orafti® P95, Degree of polymerization 2–8, Beneo Orafti; Tienen, Belgium), 20 g.L^−1^ of sodium ascorbate (Royal DSM N.V.; Limburg, Netherland) and 9 g.L^−1^ of NaCl. Two independent fermentation procedures were performed for each LAB to have two biological replicates for analysis. After resuspension in the protective medium, samples were divided into three parts: one part was directly frozen at -80 °C in Eppendorf sterile tubes to obtain the frozen samples, another one was placed in Petri dishes for freeze-drying (30 g per Petri dish, around 3–4 Petri dishes per formulation depending on the quantity of biomass obtained) and stored at -80 °C until drying and the third one was immediately spray-dried.

### Dehydration and storage

The spray-drying experiments were performed in a mini spray-dryer Büchi B-290 (Flawil, Switzerland). The inlet air temperature was 105 °C and the outlet air temperature 65 °C. Atomization was created by compressed air at a pressure of 6 bar and an air flux of 800 L.h^−1^. The nozzle diameter was 0.7 mm and the feed rate 0.156 L.h^−1^.

For the freeze-drying experiments, the protected bacterial concentrates frozen at -80 °C in sterile Petri dishes were transferred to pre-cooled shelves at -50 °C in a pilot-scale freeze-dryer (VirTis Genesis 35 L SQ EL-85, SP Scientific; Warminster, PA, USA). After a holding step of 1 h at -50 °C, the chamber pressure was decreased to 10 Pa and the shelf temperature was increased to -20 °C at 0.25 °C.min-^1^ to initiate the sublimation phase. After 40 h of sublimation, the shelf temperature was increased to 25 °C at 0.25 °C.min^−1^ to initiate the desorption phase that lasted 10 h.

The dried samples were packed in aluminium bags and stored at − 80 °C. For the analysis, freeze-dried samples were milled to a powder in a chamber of very low relative humidity (around 5%). All samples were conditioned in 1.5 mL sterile Eppendorf tubes (about 0.2 g) or plastic cups (about 0.5 g), for the measurement of biological and physico-chemical properties, respectively. Storage of dried samples was performed inside vacuum sealed aluminium bags for 7, 14 and 28 days at 4, 23 and 37 °C.

### Biological studies

#### Culturability

Frozen samples were thawed in a water bath at 37 °C for *Lpb. plantarum* WCFS1 and 42 °C for *Lb. bulgaricus* CFL1, and the dried samples were rehydrated in saline water (0.9% NaCl) to reach the same dry matter relation of the protected bacterial suspension before freeze-drying, and stirred gently for 5 min at ambient temperature. After serial dilutions, in-depth spread was performed in MRS agar (Biokar Diagnostics, France) and incubated for 24 h at 37 °C for *Lpb. plantarum* WCFS1 and 48 h at 42 °C under anaerobic conditions (GENbox96124, BioMérieux, Marcy l’Etoile, France) for *Lb. bulgaricus* CFL1 before counting. Plates containing between 30 and 300 colonies were kept for cell concentration evaluation. Results were expressed as colony forming units (CFU) in log(CFU.mL^−1^).

### Acidifying activity

Acidifying activity of the samples was measured using the Cinac System described by Meneghel et al. (2017) with some modifications. A volume of 100 µL of *Lb. bulgaricus* CFL1 or 10 µL of *Lpb. plantarum* WCFS1 cell suspensions or rehydrated samples were incubated in skim milk at 42 °C or in yeast extract (10 g.L^−1^) medium supplemented with glucose (10 g.L^−1^) at 37 °C for each bacteria strain, respectively. The time necessary to reach a pH drop of 1.5 points (t_dpH=1.5_, in min) was used to characterize the acidifying activity of the samples. The longer the time, the lower the acidifying activity.

The acidifying activity results were then weighted by the culturability data to obtain the specific acidifying activity (t_spe_, in min.(log(CFU.mL^−1^)^−1^) as shown in Eq. (1) according to Gautier et al. (2013).

1 $${t}_{spe}= \frac{{t}_{dpH=1.5}}{\text{log}CFU.{mL}^{-1}}$$

Culturability and acidifying activity were measured in the protected cells before and after freezing at -80 °C, after drying, and during storage of the dried samples after 7, 14 and 28 days. Since the statistical analysis demonstrated that no loss of culturability nor acidifying activity occurred because of the freezing operation (Table SM1), the frozen samples were used for further analyses and considered as the reference condition to evaluate the effect of the different stabilization and storage scenarios.

Then, the t_spe_ was plotted as a function of the storage time (t, in days) and the rate of loss of acidifying activity (k_spe_, in min.(log(CFU.mL^−1^))^−1^.day^−1^) was determined from the slope of the curve for each storage temperature according to Eq. 2 .

2 $${t}_{spe}= {k}_{spe}\times t+A$$

Subsequently, the loss rate was correlated with the inverse of the storage temperature (in K^−1^) and fitted to the Arrhenius equation following Eq. 3 :

3 $${k}_{spe}=A\times {e}^{{~}^{-Ea}\!\left/ \!{~}_{R\times T}\right.}$$

Where A is a frequency factor (in days^−1^), Ea is the apparent energy of activation (in kJ.mol^−1^), R is the gas constant (8.314 J.K^−1^.mol^−1^), and T is the storage temperature (in K). From the slope of the linearization of  Eq. 3 (Eq. 4) the Ea can be computed (Sosa et al. 2016).

4 $$\text{ln}{k}_{spe}= -{~}^{{E}_{a}}\!\left/ \!{~}_{R T}\right.+ \text{ln}A$$

### Membrane integrity

Flow cytometry analyses with propidium iodide (PI) and carboxyfluorescein diacetate (cFDA) dual staining were carried out to quantify viable, injured, and dead cells. PI can only enter the cell and bind DNA if membrane damage has occurred, giving information on cell membrane integrity. cFDA only dyes cells that are still viable and gives information on intracellular esterase activity (Rault et al. 2007). A volume of 100 µL of bacterial suspension (thawed or rehydrated cells) containing approximately 10^7^ cells.mL^−1^ was added to 900 µL of McIlvaine’s buffer (pH 7.4). The markers consisted of 10 µL of Chemchrome V8 (Bio-Mérieux, Craponne, France) diluted to 1/10 (v/v) in acetone (Sigma Aldrich, Saint Quentin Fallavier, France) and 5 µL of PI (10 mg PI, Sigma, diluted in 10 mL H_2_O MilliQ), added to one millilitre of the diluted bacterial suspension containing approximately 10^6^ cells.mL^−1^. The suspension was then incubated for 10 min at 40 °C before flow cytometry analysis.

The experiments were performed with a CyFlow Space cytometer (Sysmex-Partec, Villepinte, France) equipped with a solid blue laser, emitting at 488 nm and four optic filters, following the set-up described by Bouix et al. (2022). Data were collected with FloMax software (SYSMEX-PARTEC, Villepinte, France) and the numbers and percentages of stained cells determined by each detector were analyzed.

### Physical properties

#### Water activity determination

The water activity (a_w_) of the dried samples was measured at 25 °C using a a_w_ meter labMasteraw (Novasina, Precisa, Poissy, France).

### Glass transition temperature

Glass transition temperatures were determined on the frozen cells (T_g_’, in °C) as well as on the freeze- and spray-dried powders (T_g_, in °C) before storage and after 28 days of storage by differential scanning calorimetry (DSC) following the calibration and measurement procedures described by Guerrero Sanchez et al. (2022) with slight modifications. A Diamond equipment with a liquid nitrogen cooling accessory (CryoFill, Perkin Elmer) was used to analyze the frozen samples that exhibited thermal events at negative temperatures; while a Pyris 1 equipment with a mechanical cooling system (Intracooler 1P, Perkin Elmer) was used for the dehydrated samples (both Perkin Elmer LLC, Norwalk, CT, USA). Approximately 10 to 20 mg of each sample were placed in 50 µL hermetically sealed aluminium pans. An empty pan was used as a reference. Linear heating and cooling rates of 10 °C.min^−1^ were used. Freeze-thawed samples were cooled to -100 °C and then scanned up to 20 °C. For the dehydrated samples a first heating step from 5 to 110 °C was performed to erase their thermal history, followed by cooling to 5 °C and a second heating up to 130 °C. The glass transition temperature was determined from the maximum of the first derivative of the second heating curve.

### Biochemical properties by FTIR micro-spectroscopy

Frozen samples were thawed, and dried samples were rehydrated as described in previous sections. After thawing or rehydration, the suspensions were washed three times with saline water (0.9%) and centrifuged at 16,000 g for 10 min at 4 °C. Thermal source transmission FTIR micro-spectroscopy measurements were performed following the procedure described by Guerrero Sanchez et al. (2022) and Meneghel et al. (2020). A total of between 60 and 80 spectra were recorded for each sample.

### Pre-processing of raw spectra

Spectra showing Mie scattering or absorption features were sorted using the Omnic software. Then an automatic atmospheric subtraction was applied to all spectra to remove the residual contribution from water vapour and carbon dioxide.

### Procedure for water subtraction

The subtraction of bulk water contributions form spectra of bacteria was performed by using an in-house MATLAB script (version: 8.3.0.532, MathWorks, Natick, MA, USA) according to the procedure described by Meneghel et al. (2020). To calibrate the water removal program, the value of the amide I to amide II area ratio was used. The amide I area was calculated between 1727 and 1585 cm^−1^ and the amide II between 1585 and 1481 cm^−1^ with a baseline extending from 1727 to 1481 cm^−1^.

### Post-processing of spectra

Post-processing of the water-subtracted spectra was performed using the Unscrambler®X software package (Version 10.2, CAMO Software AS, Oslo, Norway). The analysis focused on three spectral regions: (1) from 3060 to 2800 cm^−1^, containing information on lipids, mainly fatty acyl chains of the bacterial membrane; (2) from 1800 to 1370 cm^−1^ to recover information about proteins, particularly amide I and amide II bands; and (3) from 1369 to 975 cm^−1^, a complex region containing information on proteins (amide III band), nucleic acids, and cell wall components such as phosphorylated molecules and polysaccharides. Each spectra region was normalized and baseline was corrected using the extended multiplicative scatter correction (EMSC). The post-processed data was statistically analyzed by principal component analysis (PCA) to study the variation pattern by an unsupervised method using the Unscrambler®X software package.

To deepen the analysis, and according to Guerrero Sanchez et al. (2022, 2023), the second-order derivatives of spectra were calculated to resolve protein secondary structures, identify precise peak locations, and determine the relative variation (r_υ_) of the peak’s height in the dried sample compared to the frozen sample. The assignment of the principal absorption bands was performed using data from the literature.

### Statistical analysis

Nonparametric Kruskal-Wallis tests were performed over biological activity and the glass transition temperature measurements using XLSTAT 19.6 (Addinsoft, Paris, France). ANOVA analyses were carried out on biochemical composition determined by FTIR spectroscopy data. A significance level of 95% (*p* < 0.05) was considered in all cases. Three technical replicates were performed for each biological replicate except for water activity.

## Results

### Biological activity

#### Culturability and acidifying activity

To study the impact of the dehydration operation on the biological activity of the LAB, the specific acidifying activity (t_spe_) was studied before and after drying (Table 1). An increase in t_spe_ was observed after drying for all the studied samples, as a result of the loss of culturability and decrease of the acidifying activity after this operation. Spray-drying caused a bigger increase in t_spe_ for both LAB strains than freeze-drying. Also, *Lb. bulgaricus* CFL1 presented bigger t_spe_ increases for each drying method than *Lpb. plantarum* WCFS1.

**Table 1:** Specific acidifying activity (t_spe_) (in min.(log (CFU.mL^−1^)^−1^) of the frozen, freeze-dried (FD) and spray-dried (SD) samples of *Lb. bulgaricus* CFL1 and *Lpb. plantarum* WCFS1 and the increase of Δt_spe_ after drying

	t_spe_ frozen	t_spe_ SD	t_spe_ FD	Δt_spe_ SD	Δt_spe_ FD
CFL1	40.4^B^	83^D^	66^C^	42^d^	25^b^
IQR	0.7	4	1	5	1
WCFS1	30^A^	64^C^	36.9^B^	34^c^	7^a^
IQR	2	2	0.6	3	2

Data presented as median and interquartile ranges (IQR). Different superscript letters represent statistical differences between samples at a 95% confidence level: uppercase letter for absolute t_spe_ values comparison; lowercase letters for Δt_spe_ values comparison

**Fig. 2:**
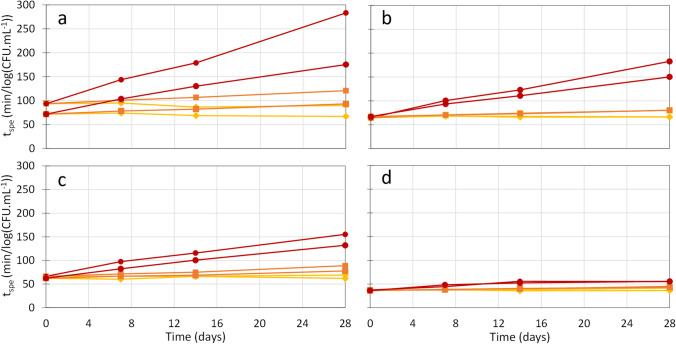
Evolution of the specific acidifying activity (t_spe_) of each biological replicate during 28 days storage at 4 °C (yellow diamonds), 23 °C (orange squares), and 37 °C (red circles). (**a**) *Lb. bulgaricus* CFL1 spray-dried sample, (**b**) *Lb. bulgaricus* CFL1 freeze-dried, (**c**) *Lpb. plantarum* WCFS1 spray-dried samples and (**d**) *Lpb. plantarum* WCFS1 and freeze-dried samples. The regression coefficients R^2^ of the linear fit for the curves at 23 and 37 °C were higher than 0.75 in all cases

**Fig. 3:**
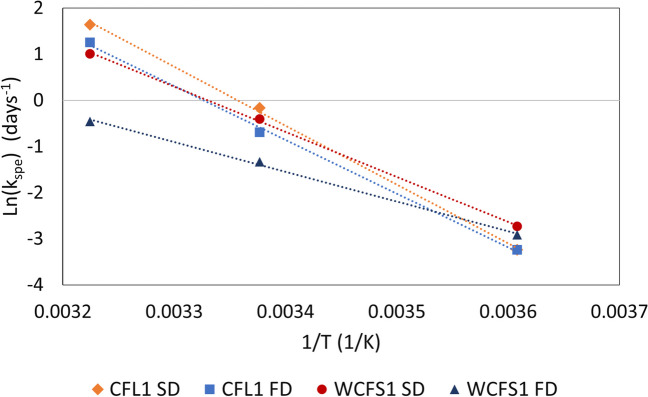
Linearization of the Arrhenius equation (Eq. 4) of the rate of specific acidifying activity loss versus the inverse of the storage temperature. The activation energy (Ea_spe_) for each sample was determined from the slope of the linear fit and is shown in Table 2

The progress of t_spe_ with the storage time for three storage temperatures is shown in Fig. 2. No significant losses were observed after 28 days of storage at 4 °C. At 23 and 37 °C, however, whatever the LAB and the drying method, a linear increase of t_spe_ with storage time was observed, following the relationship shown in Eq. 2. Linear relationships between the specific acidifying activity with storage time have also been described by other authors when analyzing different storage conditions (Passot et al. 2012; Streit et al. 2008). Table 2 shows the slopes of the linear regressions corresponding to specific acidifying activity loss rates (k_spe_) after 28 days of storage at each tested temperature for the four samples. The higher the k_spe_, the faster the bacterial inactivation. Both spray-dried and freeze-dried samples of *Lpb. plantarum* WCFS1 showed smaller inactivation rates than *Lb. bulgaricus* CFL1 ones after 28 days of storage at the three temperatures. Also, the k_spe_ values of spray-dried samples were bigger than those for freeze-dried ones (except for samples stored at 4 °C, which did not exhibit significant losses). Additionally, k_spe_ values were correlated to the absolute storage temperature and fitted to the linearized Arrhenius equation (Eq. 4) to determine the activation energy Ea_spe_ of each sample (Fig. 3; Table 2). Although the activation energy is more commonly computed with the culturability loss rate (k) rather than the specific acidifying activity loss rate (k_spe_), the activation energy values determined by this procedure were consistent with those found with the culturability results (Table SM2). Spray-dried cells of both LAB and freeze-dried ones of *Lb. bulgaricus* CFL1 showed similar activation energy values, while freeze-dried *Lpb. plantarum* WCFS1 samples showed the lowest activation energy.

**Table 2:** Specific acidifying activity loss rate (k_spe,_ in (min.(log (CFU.mL^−1^))^−1^.day^−1^) of freeze-dried (FD) and spray-dried (SD) samples of *Lb. bulgaricus* CFL1 and *Lpb. plantarum* WCFS1 during storage at 4, 23 and 37 °C and their corresponding activation energy (Ea_spe_, in kJ/mol))

	k_spe_ (min.(log (CFU.mL^−1^))^−1^.day^−1^))			Ea_spe_ (kJ/mol)	R^2^
	4 °C	23 °C	37 °C		
CFL1 SD	0.04	0.9	6	105	0.999
IQR	0.00	0.1	1	6	
CFL1 FD	0.04	0.50	3.5	97	0.998
IQR	0.01	0.03	0.6	10	
WCFS1 SD	0.07	0.7	2.7	85	0.999
IQR	0.03	0.1	0.3	11	
WCFS1 FD	0.05	0.27	0.638	41	0.998
IQR	0.05	0.02	0.007	13	

Data presented as median and interquartile ranges (IQR). The correlation coefficient R^2^ informs on the quality of the linear regression fitting used to calculate Ea_spe_

### Membrane integrity and cell viability

**Fig. 4:**
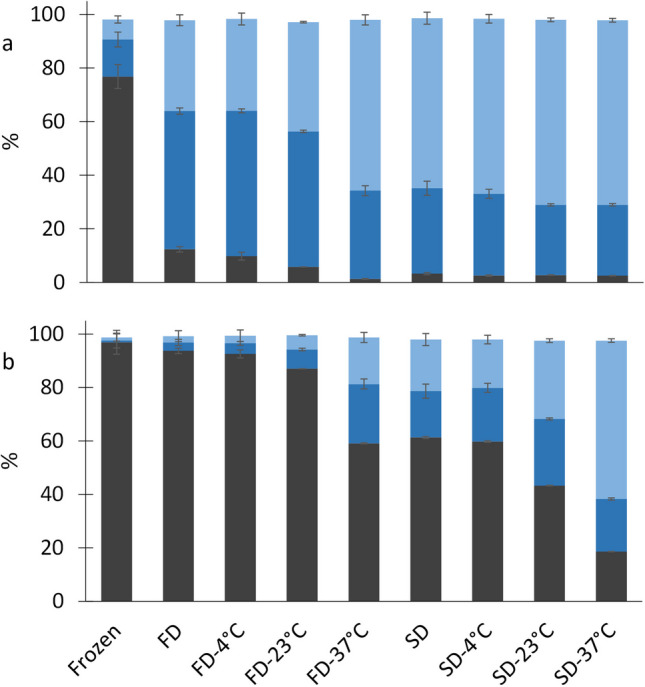
Viable (gray bars), injured (blue bars), and dead cells (light blue bars) for the frozen samples, freeze-dried (FD) and spray-dried (SD) samples before storage, and after 28 days storage at 4, 23 and 37 °C. (**a**) *Lb. bulgaricus* CFL1; (**b**) *Lpb. plantarum* WCFS1

Figure 4 shows the percentage of viable, viable and injured, and dead cells for the two LAB and the two drying methods before and after dehydration, and after 28 days of storage at the different temperatures. The results showed important losses of viability and membrane integrity for *Lb. bulgaricus* CFL1 after both drying methods (Fig. 4a), while *Lpb. plantarum* WCFS1 exhibited a higher tolerance to the dehydration step (Fig. 4b). The spray-drying operation caused the death of more than 60% and 19% of *Lb. bulgaricus* CFL1 and *Lpb. plantarum* WCFS1 populations, respectively; and it increased the percentage of injured cells to 32% and 17% for each bacteria strain, respectively. In contrast, freeze-drying maintained a much higher proportion of viable and not injured *Lpb. plantarum* WCFS1 cells (93%). Storage at 23 and 37 °C amplified the damages caused after drying, increasing the percentage of dead and injured cells, except for the *Lpb. plantarum* WCFS1 freeze-dried samples, that maintained a low proportion of dead cells after 28 days of storage at 23 °C (5%) and even at 37 °C (17%).

### Physical properties

**Fig. 5:**
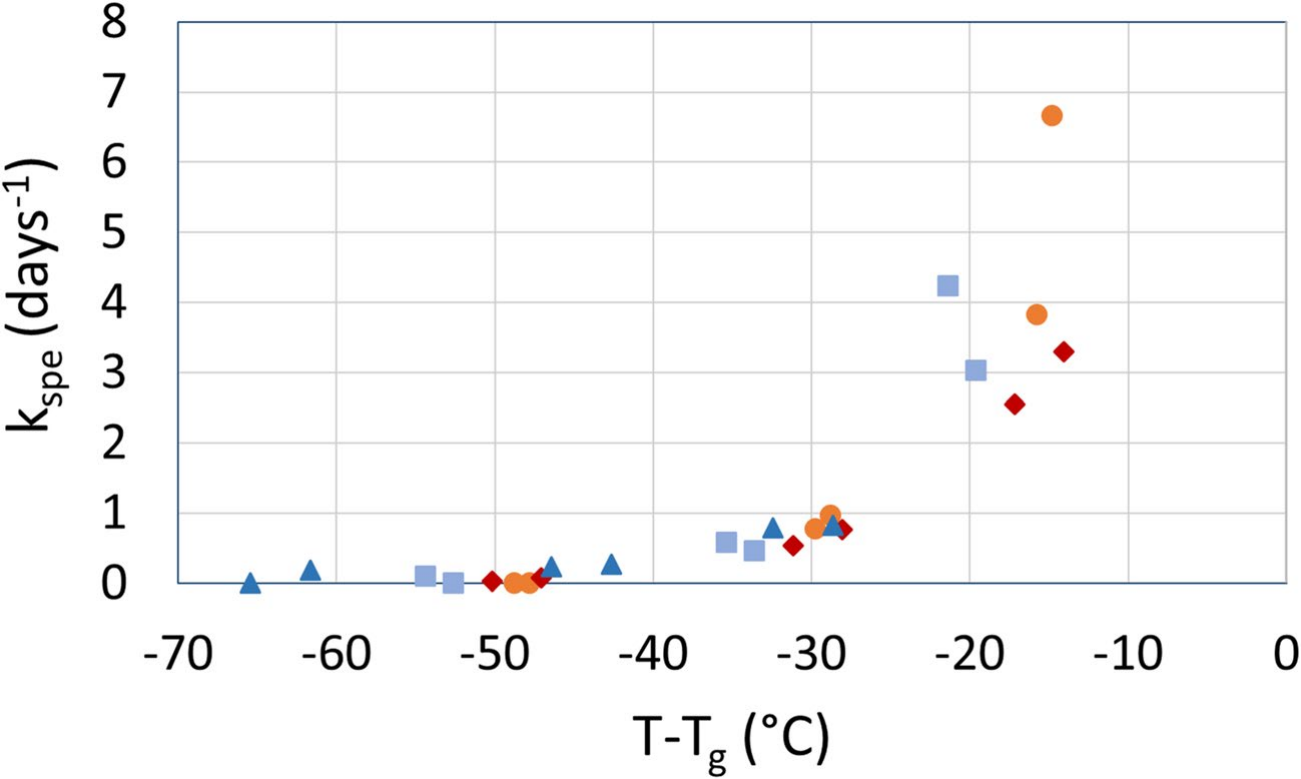
Rate of loss of specific acidifying activity (k_spe_) as a function of (T-T_g_) for spray-dried *Lb. bulgaricus* CFL1 (orange circles), freeze-dried *Lb. bulgaricus* CFL1 (light blue squares), spray-dried *Lpb. plantarum* WCFS1 (red diamonds) and freeze-dried *Lpb. plantarum* WCFS1 (blue triangles)

Table 3 shows the glass transition temperature of the maximally concentrated phase (T_g_’) of the frozen samples and the glass transition temperature (T_g_) and water activity (a_w_) of the dehydrated samples before storage. As expected, no significant differences were observed between the T_g_’ of the two frozen bacterial suspensions since a unique protective solution, and in the same proportion was used for both samples. Freeze-dried samples showed half lower a_w_ values than spray-dried ones, which resulted in higher T_g_ values. Also, *Lpb. plantarum* WCFS1 dried samples exhibited a lower water activity than *Lb. bulgaricus* CFL1, and a higher T_g_ of the freeze-dried sample. Different drying batches and cell dehydration-rehydration properties could explain the differences observed among the strains. To evaluate the impact of the restricted mobility of the matrix in the glassy state on the specific acidifying activity loss rate, k_spe_ was plotted as a function of the difference between the storage temperature and the glass transition temperature (T-T_g_) (Fig. 5). For (T-T_g_) values under − 40 °C k_spe_ values were close to zero. As the (T-T_g_) parameter increased from − 40 to -20 °C the k_spe_ slightly increased. When the (T-T_g_) difference raised above − 20 °C a more abrupt increase of the k_spe_ was observed.

**Table 3:** Glass transition temperature of the maximally concentrated phase (T_g_’) of the frozen samples and the glass transition temperature (T_g_) and water activity (a_w_) of the dehydrated samples before storage

	T_g_’ *	a_w_		T_g_	
		Spray-dried	Freeze-dried	Spray-dried	Freeze-dried
CFL1	-29.1	0.174^d^	0.088 ^b^	52.3^a^	57.5^b^
IQR	1.7	0.003	0.002	0.5	0.9
WCFS1	-29.5	0.141^c^	0.033 ^a^	52.6^a^	67.5^c^
IQR	0.1	0.003	0.004	1.9	1.5

Data presented as median and interquartile ranges (IQR). Different superscript letters within each method represent statistical differences between samples at a 95% confidence level

* Three analytical replicates of only one biological replicate were measured for this determination

#### Biochemical properties of cells by FTIR micro-spectroscopy

**Fig. 6:**
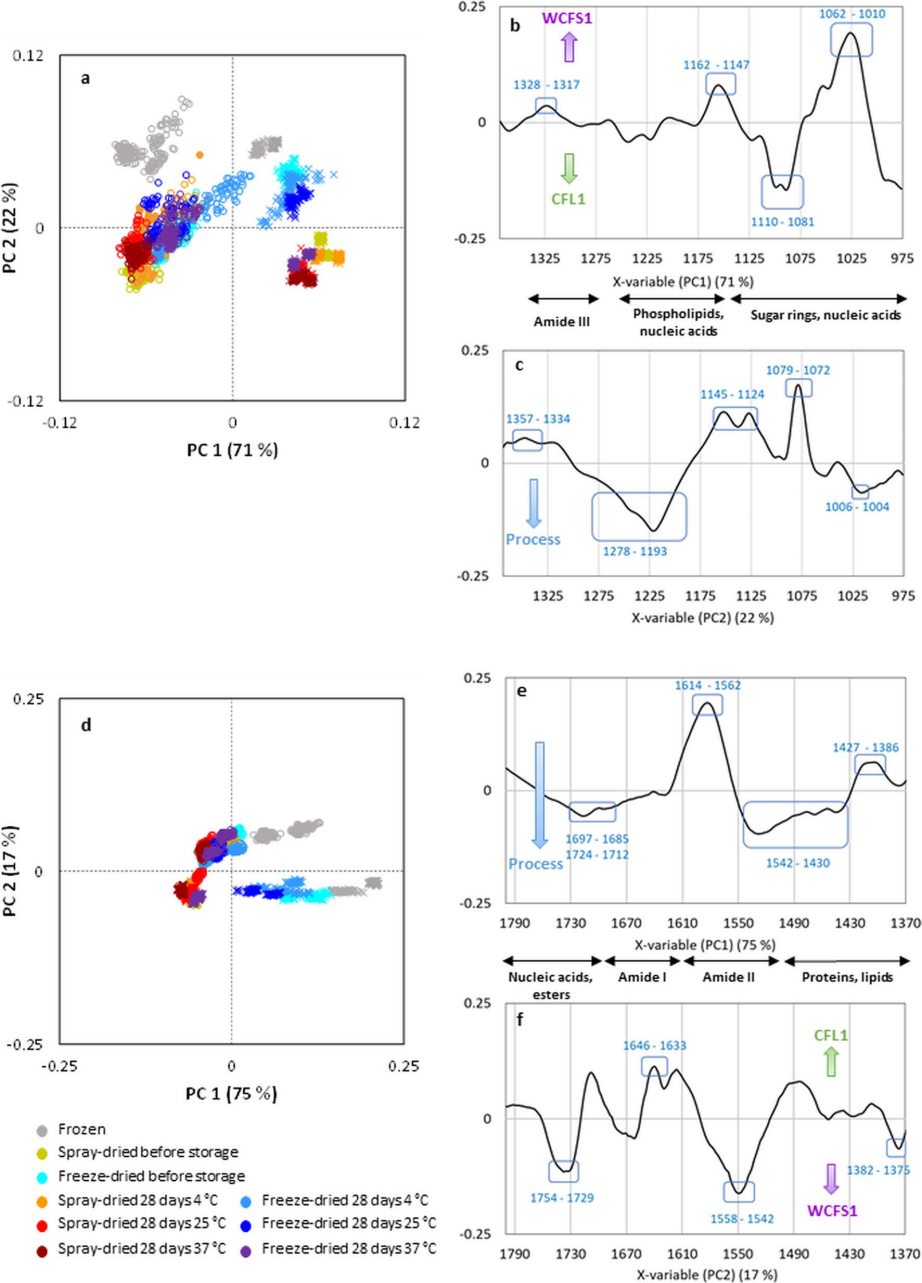
Principal component analysis (PCA) of the FTIR normalized and corrected spectra of the of *Lb. bulgaricus* CFL1 (circles) and *Lpb. plantarum* WCFS1 (crosses) frozen, freeze-dried and spray-dried samples, before storage and after storage for 28 days at 4, 23 and 37 °C; in the spectral regions 1369 –975 cm^−1^ (**a**, **b** and **c**) and 1800 –1370 cm^−1^ (**d**, **e** and **f**). Both biological replicates are plotted independently in the score plots (**a** and **d**) and the discriminating bands are indicated in the loading plots (**b** and **e** for PC1; **c** and **f** for PC2)

Principal component analyses (PCA) were performed in the three regions of interest for both biological replicates of the frozen, freeze-dried and spray-dried bacterial concentrates, before storage and after storage for 28 days at 4, 23 and 37 °C. In Fig. 6 and SM1 the PC1 vs. PC2 plots are displayed with their corresponding loadings. The analysis clearly discriminated between *Lb. bulgaricus* CFL1 and *Lpb. plantarum* WCFS1 bacterial populations in the three spectral regions. As displayed in Fig. 6, for *Lb. bulgaricus* CFL1 (circles) two separated clusters were observed that grouped the frozen and the dehydrated samples. Whereas for *Lpb. plantarum* WCFS1 (crosses), it was also possible to discriminate between the freeze- and the spray-dried samples.

The spectral region between 1369 and 975 cm^−1^ gives interesting information on the amide III band of proteins, nucleic acids, and phosphorylated molecules and polysaccharides from the cell wall. In this region, PC1 and PC2 explained 93% of the total variance (Fig. 6a). PC1 (71% of variance) discriminated both LAB. *Lpb. plantarum* WCFS1 cells were described by positive scores associated with bands at 1328 –1317 cm^−1^ (amide III bands of proteins), 1162 –1147 cm^−1^ (proteins from the cytoplasm and membranes, nucleic acids and sugar rings from the cell wall) and 1062 –1010 cm^−1^ (mostly peptidoglycan from the cell wall and nucleic acids). On the contrary, *Lb. bulgaricus* CFL1 cells were characterized by negative scores related to the band at 1110 –1081 cm^−1^ (carbohydrates of the membrane and cytoplasm, nucleic acids and phosphorylated proteins) (Fig. 6b). PC2 (22% of variance) showed differences between samples subjected to different process severity. Positive contributions of the bands at 1357 –1334 cm^−1^ (amide III band of proteins), 1145 –1124 cm^−1^ (carbohydrates of the membrane and cytoplasm, sugar rings of the cell wall and nucleic acids) and 1079 –1072 cm^−1^ (phosphodiesters, phospholipids, and nucleic acids) were mainly associated with *Lpb. plantarum* WCFS1 frozen and freeze-dried samples and *Lb. bulgaricus* CFL1 frozen samples. Negative values at 1278 − 1193 (phosphodiesters, phospholipids, and nucleic acids) and 1006 –1004 cm^−1^ (nucleic acids) were related to *Lpb. plantarum* WCFS1 spray-dried samples and freeze-dried samples stored at 37 °C as well as a good proportion of *Lb. bulgaricus* CFL1 dehydrated samples (Fig. 6c) (Guerrero Sanchez et al. 2022, 2023; Meneghel et al. 2020; Naumann et al. 2006).

The region between 1800 and 1370 cm^−1^ is mostly related to protein components of the cell. In this spectral region the first two PCs explained 92% of the total variance (Fig. 6d). The two LAB were discriminated by PC2 (17% of variance), while PC1 (75% of variance) discriminated the samples according to different processing and storage conditions. Frozen and freeze-dried *Lpb. plantarum* WCFS1 samples before and after storage at 4 and 23 °C, as well as *Lb. bulgaricus* CFL1 frozen samples presented positive PC1 scores, due to the contribution of bands at 1614 –1562 cm^−1^ (amide I and amide II bands of proteins) and 1427 –1386 cm^−1^ (carbohydrates of the cytoplasm and membranes, amino acids from ribosomes and other proteins and nucleic acids) (Fig. 6e). All the spray-dried samples and samples stored at higher temperatures showed negative values that were related to negative bands at 1724 to 1685 cm^−1^ (amide I overlapped with esters, carboxylic acids and nucleic acids) and 1542 –1430 cm^−1^ (amide II bands of proteins and other vibrations of proteins from the cytoplasm and membranes) in the loading plot. On the other hand, for PC2, bands at 1646 –1633 cm^−1^ (β-pleated sheet structures of amide I) contributed to the positive scores of *Lb. bulgaricus* CFL1 samples, while bands at 1754 − 1729 (esters of the membrane lipids), 1558 − 1542 (amide II band of proteins) and 1382 –1375 cm^−1^ (proteins of the membranes and peptidoglycan from the cell wall) contributed to the negative ones for *Lpb. plantarum* WCFS1 (Fig. 6f) (Guerrero Sanchez et al. 2022, 2023; Meneghel et al. 2020; Naumann et al., 2006).

Finally, the region between 3060 and 2800 cm^−1^ is associated with the fatty acyl chain of membrane lipids. In this region, the variance was much smaller compared to the other two regions and the first two PCs only explained 57% of the total variance (Fig. SM1.A). In this region signals related to the fatty acids chain of membrane lipids (2925–2937 and 2858 –2846 cm^−1^) contributed to PC1’s negative scores associated with most of *Lb. bulgaricus* CFL1 samples (except for one biological replicate of the frozen and the spray-dried sample stored at 23 °C); while signals possibly related to cyclopropane ring of cyclic FA (2995 –2975 cm^−1^) and other FA chain of membrane lipids (2941 –2937 cm^−1^) contributed to the positive scores linked to *Lpb. plantarum* WCFS1 (Fig.SM3.B) (Guerrero Sanchez et al. 2023; Kochan et al. 2018).

**Fig. 7:**
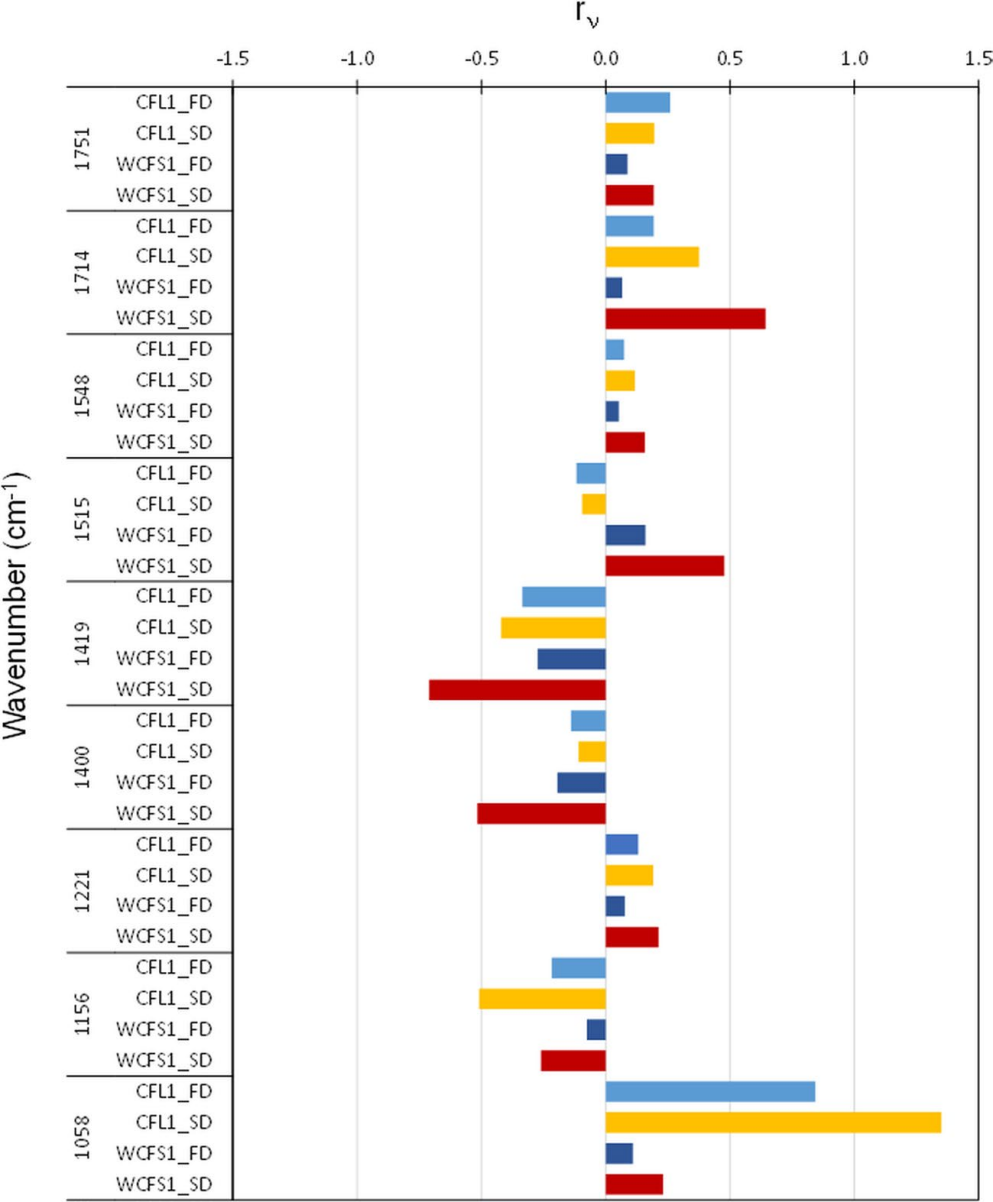
Changes in the peaks’ height (r_v_) of the mean second derivative of the FTIR bands after freeze- or spray-drying compared to their frozen sample. Only peaks that showed changes larger than 15% are displayed

The second-order derivative of the spectra (Fig. SM2)  made possible the identification and assignment of the peaks to specific components of the cells according to the bibliography (Table SM3). The relative variation of the peaks’ height after drying compared to the frozen samples (r_v_) was calculated and the bands corresponding to the wavenumbers that suffered a variation larger than 15% for at least one of the samples are displayed in Fig. 7. A positive value means an increase of the peak’s height and *vice-versa*. In the 1800 –1370 cm^−1^ region, the drying step caused important increases of the bands at 1751 cm^−1^ (νC = O of esters) for all the samples (smaller change for the freeze-dried sample of *Lpb. plantarum* WCFS1), the bands at 1714 (νC = O, νC = N, νC = C and δNH of esters, carboxylic acids and nucleic acids of DNA/RNA and ribosomes), 1548 ((δN-H) + (νC-N) of amide II) for the spray-dried samples of *Lpb. plantarum* WCFS1 and *Lb. bulgaricus* CFL1, and 1515 cm^−1^ (νCC and δCH of amino acids, particularly tyrosine, of the membrane and cytoplasm), especially for the spray-dried samples of *Lpb. plantarum* WCFS1. On the other hand, a decrease of the bands at 1419 (δC-O-H of carbohydrates, proteins and nucleic acids of membrane, cytoplasm, nucleoid and ribosomes, or νC-O sym of COO^-^ of phospholipids from the membrane) and 1400 cm^−1^ (νC = O sym of COO^-^ and νCOO^-^ sym of amino acids, fatty acid and peptidoglycan of the membrane, cytoplasm and cell wall) was observed for these samples. In the 1370 –975 cm^−1^ region, the dehydration step caused an increase of the band at 1058 cm^−1^ (νCO, νCC, δOCH, νPO2-, νC-OH, νC-O-C sym and νP-O-C sym of phospholipid phosphate, oligosaccharides, polysaccharides, sugar rings, pectin and peptidoglycan and deoxyribose of the membrane, cytoplasm, nucleoid and cell wall) and a decrease of the band at 1156 cm^−1^ (νC–O of proteins, carbohydrates and nucleic acids of the membrane and cytoplasm) more pronounced for *Lb. bulgaricus* CFL1 and the spray-dried samples. In general, spray-drying caused bigger changes in the peaks’ height than freeze-drying except for the bands at 1751, 1515 and 1400 cm^−1^ for *Lb. bulgaricus* CFL1 cells.

## Discussion

Most research on the effect of drying on LAB cannot be compared due to varying fermentation conditions, protection and/or stabilization strategies. The storage conditions often also differ or are not investigated. Moreover, different bacteria may exhibit different resistance to a drying process. The present article thus aimed to investigate the mechanisms underlying the preservation/degradation of LAB functionalities (culturability and acidifying activity) during drying and storage. For this purpose, two strains exhibiting different sensitivity were protected with the same protective solution and exposed to freeze- or spray-drying and to relevant storage temperatures for dried bacteria. Diverse techniques, from flow cytometry to FTIR spectroscopy and DSC, were used to analyze their biochemical and physical properties. Our results suggest the osmotic and mechanical stresses, occurring when freeze- or spray-drying, as the principal causes of damage to *Lb. bulgaricus* CFL1, and the thermal stress associated with spray-drying as the main stress affecting *Lpb. plantarum* WCFS1. This study also showed that, while the sensitivity of *Lb. bulgaricus* CFL1 to freeze-drying can be ascribed to loss of membrane integrity and cell wall degradation, heat inactivation of both strains associated with spray-drying can be explained by the modification of nucleic acids and proteins. The present results also substantiated the importance of the physical properties of the glassy matrix in the loss of activity rates, confirming that a glass transition temperature 40 °C lower than the storage temperature should be reached to preserve cells during storage.

The higher resistance of *Lpb. plantarum* WCFS1 compared to *Lb. bulgaricus* CFL1 was expected from previously reported work on the environmental stress responses of such subspecies (Arena et al. 2019; Capozzi et al. 2012; Mille et al. 2005; Passot el al., 2012, others). Moreover, while *Lb. bulgaricus* CFL1 was severely damaged by both drying methods, *Lpb. plantarum* WCFS1 exhibited less than three times lower loss of functionality after freeze-drying than *Lb. bulgaricus* CFL1 (Δ_tspe_ FD 7 and 25 min.(log (CFU.mL^−1^), respectively). This could be a consequence of the different biochemical composition between both strains induced by fermentation, as seen in the PCA results of the FTIR measurements, where both LAB exhibited important differences in the PC1 of the regions associated with cell wall components and PC2 of the region related to proteins. By considering each drying method as a combination of three main stresses: osmotic mechanical and heat, it was possible to propose that *Lpb. plantarum* WCFS1 can tolerate better the osmotic and mechanic stress involved in both dehydration processes but is sensitive to the heat stress caused by spray-drying only. *Lb. bulgaricus* CFL1 is, in turn, sensitive to all three stresses, but certainly less sensitive to heat than *Lpb. plantarum* WCFS1. Our hypothesis agrees with previous work by Perdana et al. (2013), where from experimental and modelling studies at different inlet air temperatures, the authors concluded that the viability loss of *Lpb. plantarum* WCFS1 at high temperatures could be explained entirely by thermal inactivation. The differentiation between thermal and dehydration inactivation proposed in the present study also agrees with previous work by Lievense et al. (1992), which evidenced that thermal inactivation of *Lpb. plantarum* becomes relevant when temperatures are higher than 50/55°C on fluidised bed drying. According to Peighambardoust et al. (2011), the outlet air temperature is a crucial parameter, which depends on many operating variables and has a major influence on the viability of spray-dried starter cultures.

The membrane integrity measurements confirmed the cytoplasmatic membrane as a site of damage during drying, as recognized by other authors for cells facing freeze-drying (Velly et al. 2015) and spray-drying (Huang et al. 2017). The high loss of membrane integrity observed for *Lb. bulgaricus* CFL1 following freeze-drying, and the comparatively lower effect of heat suggests a predominance of osmotic and mechanical effects of dehydration, inducing membrane injury. On the other hand, the percentage of injured and dead *Lpb. plantarum* WCFS1 cells significantly increased only with the temperature increase of the drying process and storage, thus revealing that heat stress also caused some membrane damage. The substantial contrast in the membrane resistance between the two strains against freeze-drying implies differences in the membrane organization that may provide *Lpb. plantarum* WCFS1 with a robust membrane little affected by osmotic and mechanical stresses.

The cell damage during drying and storage can be related to cell biochemical modifications evaluated by FTIR micro-spectroscopy. The differences observed for the two microorganisms were not only restricted to components of the cell membrane, but were also related to their nucleic acids, protein, and cell wall constituents. Recent works have also highlighted the modifications of many of these other cell constituents during drying processes (Romano et al. 2021; Guerrero Sanchez et al. 2022, 2023). In the present work, while the spectral region related to lipidic components showed only moderate variations, the largest variance was observed in the 1800 to 1370 cm^−1^ and 1369 and 975 cm^−1^ regions. Therefore, the loss of functionality during drying and storage was mainly associated with damage to nucleic acids and proteins (Figs. 6 and 7). In particular, the bands related to nucleic acids (νC = O 1714 and 1221 cm^−1^ associated with asymmetric stretching P = O), proteins (amide II at 1548 cm^−1^ and tyrosine at 1515 cm^−1^), and complex vibrations of lipids and proteins (C-O-H and stretching COO^−^ at 1419 and 1400 cm^−1^) showed much bigger changes after spray-drying of *Lpb. plantarum* WCFS1 than after freeze-drying and may hold relevant information on the sensitivity to heat stress. Our results agree with a differential scanning calorimetry study of the damage induced by heat over *Lb. bulgaricus* cells that showed damage to the cytoplasmic membrane at temperatures below 64 °C, while at temperatures of 65 °C and above, the cell wall and proteins (specifically ribosomes) were critical sites of injury (Teixeira et al. 1997). Even though *Lb. bulgaricus* CFL1 also exhibited modifications in nucleic acid bands and proteins, the main modifications were observed in cell wall carbohydrates vibration bands (1156, 1058 cm^−1^) for both drying techniques. These bands could thus be proposed as markers of the osmotic and mechanical stress associated with dehydration and used for characterizing the most sensitive strain. In agreement with the present work, the cell envelope of *L. helveticus* was identified as a site of injury (both by FTIR and AFM), together with the nucleic acids (DNA, by FTIR) following a low-temperature vacuum drying at 43 °C (Santivarangkana et al., 2007). Our findings also support recent work highlighting the potential role of unchanged bands at 1714 and 1058 cm^−1^ (associated with nucleic acids and carbohydrates of the cell wall) as markers of *L. salivarius* (Guerrero Sanchez et al. 2022, 2023) preservation by freeze-drying (Table SM3).

The storage stability was related to the physical properties of the matrix. Since the glass transition temperature was higher than the storage temperature (T < T_g_), the protective solution ensured the immobilisation of cells in a glassy matrix. However, even in the glassy state, which is characterized by high viscosity and reduced molecular mobility, the storage stability decreased when approaching the supercooled state ((T-T_g_) approaching zero). Similar results were obtained for the viability loss of freeze-dried *L. salivarius* CECT5713 (Guerrero Sanchez et al. 2022, 2023) and *Lb. bulgaricus* (Tymczyszyn et al. 2012) as the storage temperature approached the T_g_. The increased molecular mobility, measured by ^1^H-NMR for *Lb. bulgaricus* in GOS matrices when approaching T_g_, indicated that rotational, vibrational and short movement of molecules occurs even in the glassy state (Tymczyszyn et al. 2012). Moreover, the decrease of the storage stability (increase of the rate of loss of acidifying activity) with (T-T_g_) showed similar non-linear trends for the two drying processes and was slightly more pronounced for *Lb. bulgaricus* CFL1 than *Lpb. plantarum* WCFS1 after spray-drying (Fig. 5). Wang et al. (2004) studied the viability of spray- and freeze-dried LAB during storage, and also observed higher survival rates for the freeze-dried samples than for the spray-dried ones regardless of the storage conditions. Good storage stability was achieved when cells were maintained at storage temperatures 40 °C lower than the glass transition temperatures ((T-T_g_) <-40; k_spe_ <0.3), which corresponded to samples stored at 4 °C and freeze-dried *Lpb. plantarum* cells stored at 23 °C.

The activation energy (Ea) values, calculated from the rate of loss of culturability during storage at different temperatures were consistent with those reported by Ziadi et al. (2005) for freeze-dried cells of *Lactococcus lactis* subsp. *lactis* protected with sucrose, and by Aschenbrenner et al. (2012) for freeze-dried *Lactobacillus paracasei* subsp. p*aracasei* protected with trehalose, but about two times higher than the ones informed by Sosa et al. (2016) for spray-dried *Lpb. plantarum* CIDCA83114 protected with galacto-oligosaccharides and maltodextrin mixtures (Table SM2). The Ea and Ea_spe_ values found in this work (between 47 and 85 kJ/mol, and 41 to 105 kJ/mol respectively) are, according to literature, in the range of chemically limited reactions (50 to 150 KJ/mol), while for systems controlled by diffusion processes Ea values are usually below 20 kJ/mol (Aschenbrenner et al. 2012). Aschenbrenner et al. (2012) studied the inactivation of *Lb. paracasei* and also described non-linear relationships between the viability loss rate and (T-T_g_). The authors studied the effect of different protective systems and found that, while for samples protected with dextran, the inactivation constant was solely dependent on the (T-T_g_) difference, for cells protected with di- or oligosaccharides this constant depended on both, absolute storage temperature and (T-T_g_) parameter. The authors suggest that in the first system, the key protective mechanism is the diffusion restriction due to the high T_g_ provided by the protectant, whereas in systems containing oligosaccharides water replacement may have a significant contribution towards protection. In our work, the protective agent contained maltodextrin and FOS, thus, both protective mechanisms were probably involved in the preservation of the cells during drying. This hypothesis supports the activation energy findings and explains the high rates of loss of activity even in the glassy state. Also, some reactions, such as oxidation of cell components by reactive oxygen species generated during drying do not depend strongly on diffusion and early stages of non-enzymatic browning could have occurred between the small reducing sugars of FOS and glucose and proteins remaining from the culture medium, even with diffusion limitations since in a concentrated matrix the reagents may be close enough. These reactions could also explain the biological activity loss observed even if the samples were stored in a glassy state. Moreover, *Lb. bulgaricus* CFL1 exhibited similar Ea and Ea_spe_ values in the freeze- and spray-dried samples, while spray-dried *Lpb. plantarum* WCFS1 showed a more than 50% increase of Ea compared to the freeze-dried form, thus confirming the sensitivity of this strain to increments in temperature.

In conclusion, this work evidenced the different resistance of two lactic acid bacteria to different types of stresses associated with freeze-drying and spray-drying. *Lb. bulgaricus* CFL1 appeared sensitive to osmotic, mechanical, and thermal stresses, while *Lpb. plantarum* WCFS1 showed higher resistance to the first two but appeared to be more sensitive to thermal stress associated with spray-drying. The results reached in this work enabled the identification of the cellular damages caused by spray- and freeze-drying and storage and a deeper understanding of the contribution of individual stress to the activity loss of two strains. The FTIR micro-spectroscopy method applied here in combination with flow cytometry to evidence the implication of the membrane in cell resistance to preservation and storage processes, showed that also other cell constituents such as nucleic acids, proteins, and cell wall components such as phosphorylated molecules and polysaccharides components have a fundamental influence. Nucleic acids and proteins were majorly affected by spray-drying, while the cell wall was the weakness of the more sensitive strain *Lb. bulgaricus* CFL1. Besides, the activation energy results suggested that regardless of the strain and drying process, chemically limited reactions ruled the functionality loss during storage. A more detailed study on this may be worth further analysis.

These results deliver practical guidance for improving stabilization and storage protocols. Overall, they suggested that freeze-drying of *Lpb. plantarum* WCFS1 could enable the storage of this lactic acid bacterium at mild ambient temperatures without major functionality loss. Besides, since freeze-drying and spray-drying caused a comparable degree of damage on *Lb. bulgaricus* CFL1 cells, spray-drying could be considered as a lower-cost and possibly more eco-friendly alternative, leading to practical, economic, and environmental advancements. The ongoing evaluation of the environmental performance of these two stabilization scenarios will shed light on (confirm or not) this hypothesis. The importance of the glass transition temperature and the difference between this parameter and the storage temperature (T-T_g_) on storage stability was also highlighted. (T-T_g_) values below − 40 °C guaranteed good survival while when they surpassed − 20 °C, an abrupt increase of the inactivation rate occurred. FTIR micro-spectroscopy in aqueous solutions represents a complementary and faster alternative to biological assays and could be applied for the screening of strains, fermentation, and stabilization conditions.

## Electronic supplementary material

Below is the link to the electronic supplementary material.

Supplementary file 1 (PDF 588 KB)

## Data Availability

The datasets generated and/or analysed during the current study are available in the Data INRAE repository (10.57745/AAARUZ).
