# Supplementary material for: Mechanistic study of the differences in lactic acid bacteria resistance to freeze- or spray-drying and storage
Source: Appl Microbiol Biotechnol. 2024 Jun 5;108:361. doi: 10.1007/s00253-024-13186-3 (PMC11585501; doi:10.1007/s00253-024-13186-3)
Supplement: Supplementary file 1 [file 253_2024_13186_MOESM1_ESM.pdf]

Journal: Applied Microbiology and Biotechnology

Electronic Supplementary Material

Title: Mechanistic study of the differences in lactic acid bacteria resistance to freeze- or spray-drying and storage

Authors: Maite Gagneten [a], Stéphanie Passot [b], Stéphanie Cenard [b], Sarrah Ghorbal [b], Carolina Schebor [a] and Fernanda Fonseca [b,*]

[a] Departamento de Industrias, Facultad de Ciencias Exactas y Naturales, ITAPROQ (UBA-CONICET), Universidad de Buenos Aires, Ciudad Autónoma de Buenos Aires, Argentina

[b] Université Paris-Saclay, INRAE, AgroParisTech, UMR SayFood, F-91120, Palaiseau, France

*Corresponding author: Fernanda Fonseca, Université Paris-Saclay, INRAE, AgroParisTech, UMR SayFood, F-91120 Palaiseau, France. E-mail: fernanda.fonseca@inrae.fr

The datasets generated and/or analyzed during the current study are available in the Data INRAE repository (https://doi.org/10.57745/AAARUZ).

List of tables

Table SM1. Culturability results of protected *L. bulgaricus* CFL1 and *L. plantarum* WCFS1 after production and after freezing at -80 °C 3

Table SM2. Activation energy (Ea) determined from culturability loss rate according to the Arrhenius equation for the four studied samples, and values found in the literature for the inactivation of lactic acid bacteria. 4

Table SM3. Assignment of main infrared vibrational bands of the infrared spectra identified on the studied LAB in the 3060 to 975 cm^{-1} region, according to literature. 5

List of figures

Figure SM1. Principal component analysis (PCA) of the FTIR normalized and corrected spectra of the of *L. bulgaricus* CFL1 and *L. plantarum* WCFS1 frozen, freeze-dried and spray-dried samples, before storage and after storage for 28 days at 4, 23 and 37°C; in the spectral region 3060 - 2800 cm^{-1}. (A) score plot of PC1 vs. PC2; (B) and (C) loading plots for PC1 and PC2 respectively. 9

Figure SM2. Mean second derivatives of the normalized spectrum of the frozen, freeze-dried and spray-dried cells on the (A) 3060 – 2800 cm^{-1}, (B) 1800 – 1370 cm^{-1} and (C) 1370 – 975 cm^{-1} regions. 10

Table SM1. Culturability results of protected *Lb. bulgaricus* CFL1 and *Lpb. plantarum* WCFS1 after production and after freezing at -80 °C.

	log (UFC/mL)		dtpH1.5 (min)	
	After production	Frozen	After production	Frozen
CFL1	9.65[a]	9.40[a]	369[a]	380[a]
IQR	0.12	0.03	11	8
WCFS1	11.17[b]	11.52[b]	358[a]	346[a]
IQR	0.06	0.45	8	10

Data presented as median and interquartile ranges (IQR). Different superscript letters within each method represent statistical differences between samples at a 95% confidence level.

Table SM2. Activation energy (Ea) determined from culturability loss rate according to the Arrhenius equation for the four studied samples, and values found in the literature for the inactivation of lactic acid bacteria.

Bacterial strain	Drying method	Protectant	Water activity	Ea (kJ/mol)	References
Lactobacillus delbrueckii subsp. *bulgaricus* CFL1	Spray-drying	Fructo-oligosaccharides and maltodextrin	0.174	85	This work
Lactobacillus delbrueckii subsp. *bulgaricus* CFL1	Freeze-drying	Fructo-oligosaccharides and maltodextrin	0.088	78	This work
Lactiplantibacillus plantarum WCFS1	Spray-drying	Fructo-oligosaccharides and maltodextrin	0.141	84	This work
Lactiplantibacillus plantarum WCFS1	Freeze-drying	Fructo-oligosaccharides and maltodextrin	0.033	47	This work
Lactococcus lactis subsp. *lactis*	Freeze-drying	Sucrose	Not specified	82 - 96	Ziadi et al. (2005)
Lactobacillus paracasei ssp. *paracasei*	Freeze-drying	Trehalose	0.11 – 0.22	63 - 71	Aschenbrenner et al. (2012)
Lactobacillus plantarum CIDCA83114	Spray-drying	Galacto-oligosaccharides and maltodextrin	0.22	40	Sosa et al. (2016)

References

Aschenbrenner, M., Kulozik, U., & Foerst, P. (2012). Evaluation of the relevance of the glassy state as stability criterion for freeze-dried bacteria by application of the Arrhenius and WLF model. Cryobiology, 65(3), 308–318. https://doi.org/10.1016/j.cryobiol.2012.08.005

Sosa, N., Gerbino, E., Golowczyc, M. A., Schebor, C., Gómez-Zavaglia, A., & Tymczyszyn, E. E. (2016). Effect of Galacto-Oligosaccharides: Maltodextrin Matrices on the Recovery of *Lactobacillus plantarum* after Spray-Drying. Frontiers in Microbiology, 7. https://doi.org/10.3389/fmicb.2016.00584

Ziadi, M., Touhami, Y., Achour, M., Thonart, Ph., & Hamdi, M. (2005). The effect of heat stress on freeze-drying and conservation of Lactococcus. Biochemical Engineering Journal, 24(2), 141–145. https://doi.org/10.1016/j.bej.2005.02.001

Table SM3. Assignment of main infrared vibrational bands of the infrared spectra identified on the studied LAB in the 3060 to 975 cm^{-1} region, according to literature.

Wavenumber (cm^{-1})		Assignment	Main biomolecules associated (and corresponding cellular compound or compartment)	References
This work	Literature			
1751	~1753<br>1750-1720<br>1741<br>1740-1730<br>1736	νC=O	Esters from lipids<br>(Membrane lipids)	Girardeau et al. (2022); Le Gal et al. (1991); Meneghel et al. (2020); Naumann et al. (2006); Quilès et al. (2010); Saulou et al. (2013)
1714	1716<br>1713<br>1715<br>1714<br>1713	νC=O, νC=N, νC=C, δNH	Nucleic acids, esters, carboxylic acids, (DNA/RNA, nucleoid, ribosomes)	Girardeau et al. (2022); Guerrero et al. (2022); Meneghel et al. (2020); Naumann et al. (2006); Quilès et al. (2010)
1548	1568-1531<br>1550-1563<br>1548<br>1545<br>1536<br>1530	Amide II ((δN-H) + (νC-N))	Proteins<br>(Membrane, cytoplasm)	Dianawati et al. (2012); Girardeau et al. (2022); Guerrero et al. (2022)<br>Le Gal et al. (1991); Meneghel et al. (2020); Naumann et al. (2006); Quilès et al. (2010); Santos et al. (2015); Saulou et al. (2013)
1515	1518<br>1516-1518<br>1515	νCC, δCH	Proteins, aminoacids, tyrosine<br>(Membrane, cytoplasm)	Barth et al. (2007); Guerrero et al (2022); Le Gal et al. (1991); Naumann et al. (2006); Santos et al. (2015)
1419	1415<br>1417	δC-O-H	Carbohydrates, proteins, nucleic acids (DNA/RNA)<br>(Membrane, cytoplasm, nucleoid, ribosomes)	Barth et al. (2007); Guerrero et al. (2022); Le Gal et al. (1991); Meneghel et al. (2020); Santos et al. (2015)
	1420-1405<br>1415<br>1404<br>1400	νC-O sym of COO^-	Lipids from phospholipids<br>(Membrane)	
1400	1420-1405<br>1400	νC=O sym of COO^-	Proteins (amino acids), lipids (fatty acid), carbohydrates (peptidoglycan)<br>(Membrane, cytoplasm, cell wall)	Guerrero et al. (2022); Le Gal et al. (1991); Meneghel et al. (2020);

	1401<br>1400	νCOO⁻ sym	Proteins<br>(Membrane, cytoplasm)	Naumann et al. (2006); Quilès et al. (2010)
1221	1250-1220<br>1222-1200<br>1220	νP=O asym of PO_2^-	Nucleic acids (phosphodiester of DNA/RNA), carbohydrates (teichoic and lipoteichoic acids, peptidoglycan)	Girardeau et al. (2022); Guerrero et al. (2022); Naumann et al. (2006); Quilès et al. (2010); Santos et al. (2015)
	1220	νC–O–C	Lipids of phospholipids, unsaturated fatty acids (Membrane, nucleoid, ribosomes, cell wall)	
1156	1160–1253<br>1155<br>1153	νC–O	Proteins, nucleic acids<br>(Membrane, cytoplasm)	Barth et al. (2007); Girardeau et al. (2022); Guerrero et al. (2022); Quilès et al. (2010); Saulou et al. (2013)
	1155	νC-O, νC-C,<br>C-O-C deformation	Polysaccharides, sugar rings (cell wall, peptidoglycan)	
1058	1076-1052<br>1058	νCO, νCC, δOCH, νPO_2^-, νC-OH, νC-O-C sym, νP-O-C sym	Carbohydrates (oligosaccharides, polysaccharides, sugar rings, pectin, peptidoglycan)<br>Nucleic acids (deoxyribose),<br>Lipids (phospholipid phosphate) (membrane, cytoplasm, nucleoid, cell wall)	Girardeau et al. (2022); Guerrero et al. (2022); Meneghel et al. (2020); Quilès et al. (2010)

ν = stretching, δ = bending

References Table SM3

Barth A. (2007) Infrared spectroscopy of proteins. Biochim Biophys Acta BBA - Bioenerg 1767:1073–1101 . https://doi.org/10.1016/j.bbabio.2007.06.004

Dianawati, D., Mishra, V., & Shah, N. P. (2013). Effect of drying methods of microencapsulated *Lactobacillus acidophilus* and *Lactococcus lactis* ssp. *cremoris* on secondary protein structure and glass transition temperature as studied by Fourier transform infrared and differential scanning calorimetry. Journal of Dairy Science, 96(3), 1419-1430. http://linkinghub.elsevier.com/retrieve/pii/S0022030213000635

Girardeau A, Passot S, Meneghel J, Cenard S, Lieben P, Trelea IC, Fonseca F (2022) Insights into lactic acid bacteria cryoresistance using FTIR microspectroscopy. Anal Bioanal Chem 414:1425–1443. https://link.springer.com/10.1007/s00216-021-03774-x

Guerrero Sanchez, M., Passot, S., Campoy, S. et al. Effect of protective agents on the storage stability of freeze-dried *Ligilactobacillus salivarius* CECT5713. Appl Microbiol Biotechnol 106, 7235–7249 (2022). https://doi.org/10.1007/s00253-022-12201-9

Kong, J., & Yu, S. (2007). Fourier transform infrared spectroscopic analysis of protein secondary structures. Acta biochimica et biophysica Sinica, 39(8), 549-559. https://academic.oup.com/abbs/article-lookup/doi/10.1111/j.1745-7270.2007.00320.x

Le Gal J-. M, Manfait M, Theophanides T (1991) Applications of FTIR spectroscopy in structural studies of cells and bacteria. J Mol Struct 242:397–407. https://doi.org/10.1016/0022-2860(91)87150-G

Meneghel J, Passot S, Jamme F, Lefrançois S, Lieben P, Dumas P, Fonseca F (2020) FTIR micro-spectroscopy using synchrotron-based and thermal source-based radiation for probing live bacteria. Anal Bioanal Chem 412:7049–7061 . https://doi.org/10.1007/s00216-020-02835-x

Naumann D (2006) Infrared Spectroscopy in Microbiology. In: Meyers RA (ed) Encyclopedia of Analytical Chemistry. John Wiley & Sons, Ltd, Chichester, UK, p a0117. http://doi.wiley.com/10.1002/9780470027318.a0117

Passot S, Gautier J, Jamme F, Cenard S, Dumas P, Fonseca F (2015) Understanding the cryotolerance of lactic acid bacteria using combined synchrotron infrared and fluorescence microscopies. The Analyst 140:5920–5928. https://doi.org/10.1039/C5AN00654F

Santos, M.I.; Gerbino, E.; Tymczyszyn, E.; Gomez-Zavaglia, A. (2015). Applications of Infrared and Raman Spectroscopies to Probiotic Investigation. Foods, 4, 283-305. https://doi.org/10.3390/foods4030283

Saulou, C., Jamme, F., Girbal, L. et al. (2013). Synchrotron FTIR microspectroscopy of Escherichia coli at single-cell scale under silver-induced stress conditions. Anal Bioanal Chem 405, 2685–2697. https://doi.org/10.1007/s00216-013-6725-4

Quilès F, Humbert F, Delille A (2010) Analysis of changes in attenuated total reflection FTIR fingerprints of *Pseudomonas fluorescens* from planktonic state to nascent biofilm state. Spectrochim Acta A Mol Biomol Spectrosc 75:610–616. https://doi.org/10.1016/j.saa.2009.11.026

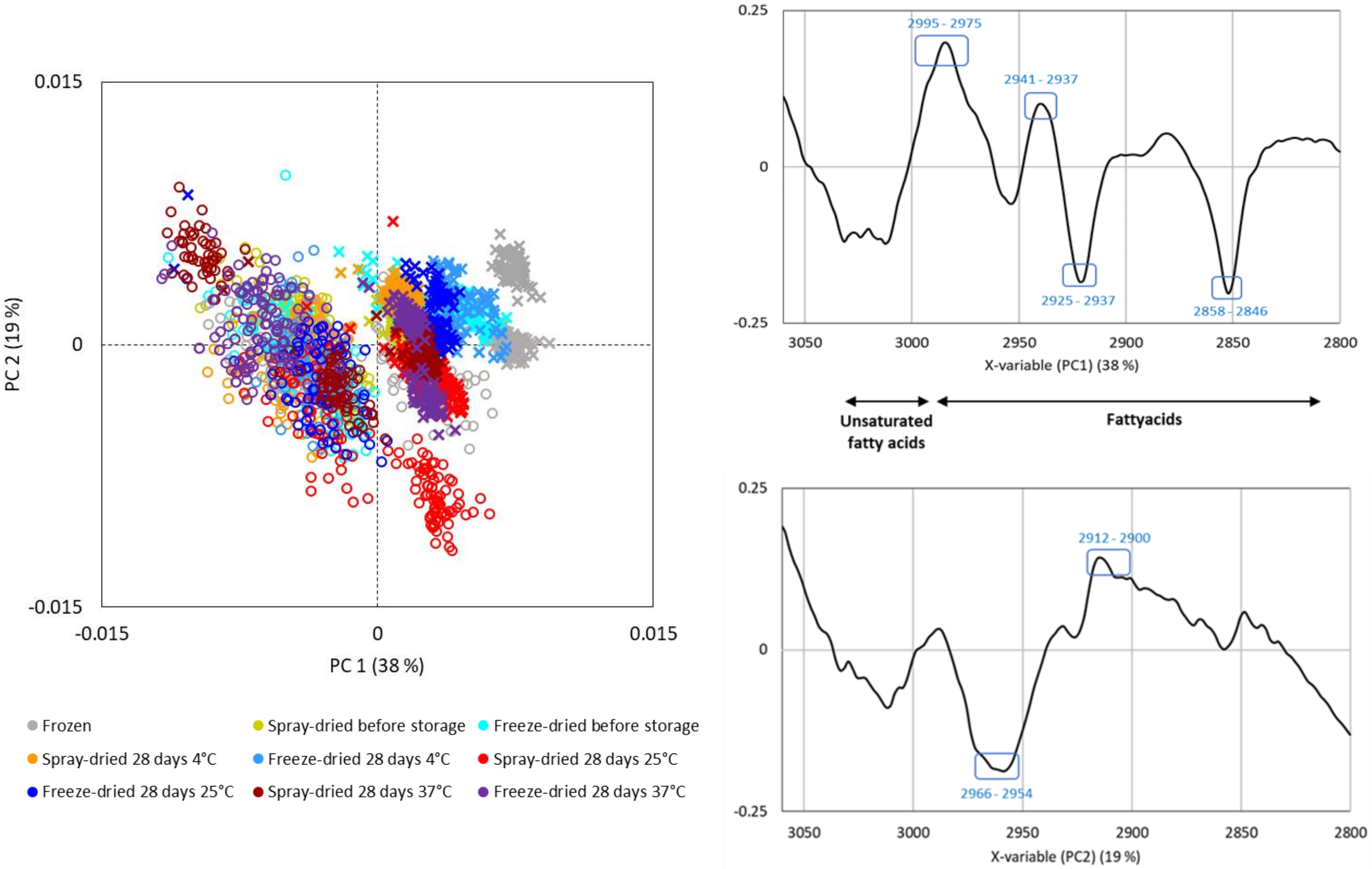

Figure SM1. Principal component analysis (PCA) of the FTIR normalized and corrected spectra of *Lb. bulgaricus* CFL1 (circles) and *Lpb. plantarum* WCFS1 (crosses) frozen, freeze-dried and spray-dried samples before storage and after storage for 28 days at 4, 23 and 37°C, in the spectral region 3060 - 2800 cm^{-1}. (A) score plot of PC1 vs. PC2; (B) and (C) loading plots for PC1 and PC2 respectively. Both biological replicates are plotted independently.

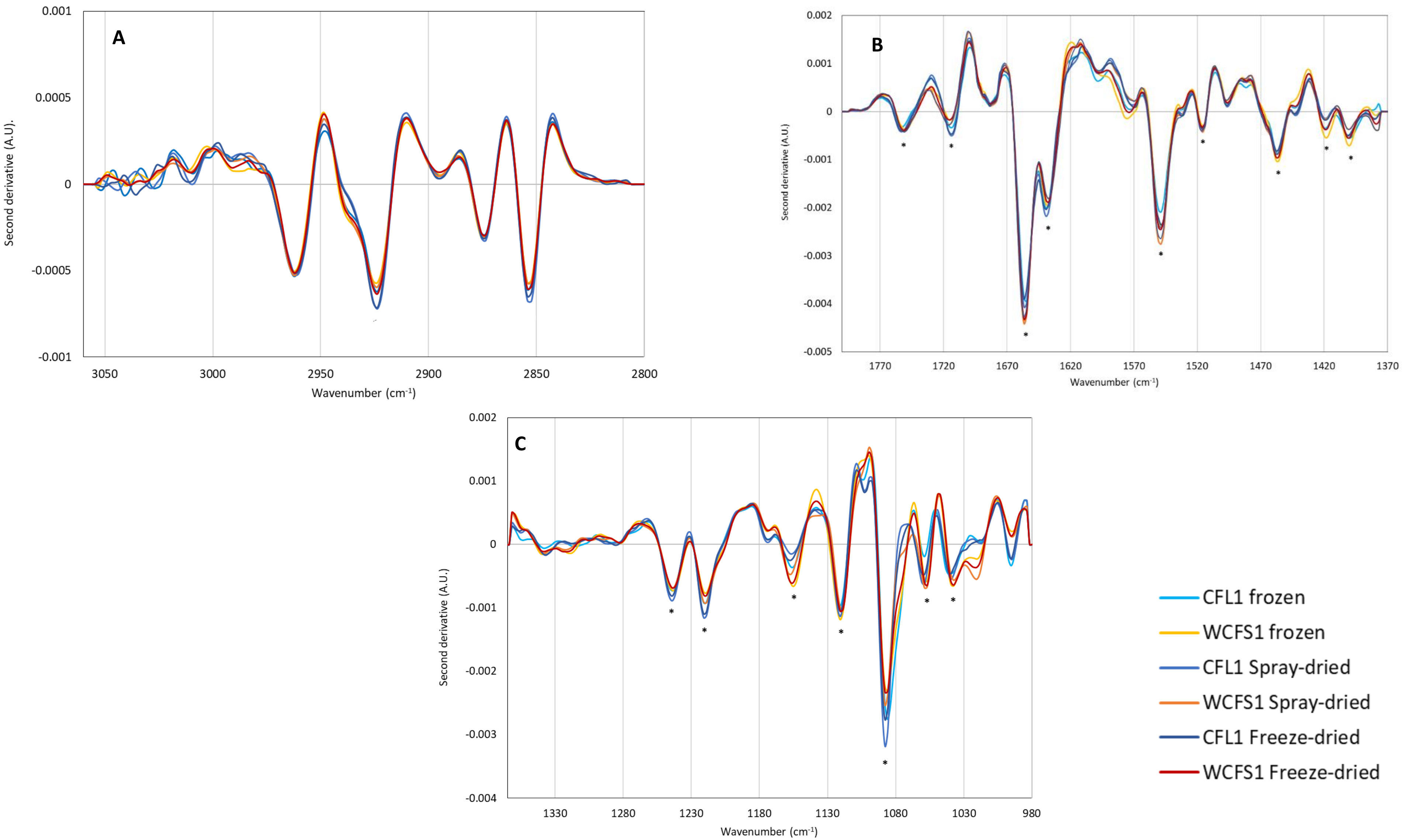

Figure SM2. Mean second derivatives of the normalized spectrum of the frozen, freeze-dried and spray-dried cells on the (A) 3060 – 2800 cm^{-1}, (B) 1800 – 1370 cm^{-1} and (C) 1370 – 975 cm^{-1} regions. The asterisks show the peaks evaluated in terms of peak height changes before and after drying.
